# Critical Assessment of Metagenome Interpretation Enters the Second Round

**DOI:** 10.1128/mSystems.00103-18

**Published:** 2018-07-10

**Authors:** Andreas Bremges, Alice C. McHardy

**Affiliations:** a Computational Biology of Infection Research, Helmholtz Centre for Infection Research, Braunschweig, Germany; b German Centre for Infection Research (DZIF), Braunschweig, Germany

## EDITORIAL

Bioinformatic methods are key components in the analysis of large omics data sets now routinely generated in microbiome research. Methods are evaluated using a large variety of benchmark data sets and evaluation metrics in original research articles, and self-assessments may be biased, which complicates method comparisons from the literature (1). CAMI, the initiative for the Critical Assessment of Metagenome Interpretation, aims to develop community-wide standards and facilitates benchmarking by providing realistic standard data sets and organizing community challenges on a regular basis. It engages the microbiome research community in public workshops and hackathons to design future CAMI challenges and identify the most relevant evaluation metrics. The first CAMI challenge opened in 2015 and ran for 3 months. Developers could assess metagenome assembly, genome and taxonomic binning, as well as taxonomic profiling methods on metagenome benchmark data sets of different complexities, derived entirely from organisms not present in public genome databases (2). All data are available for further benchmarking (https://doi.org/10.5524/100344), together with open-source software to facilitate performing and reproducing evaluations (http://microbiome-cosi.org/cami/resources).

**FIG 1:**
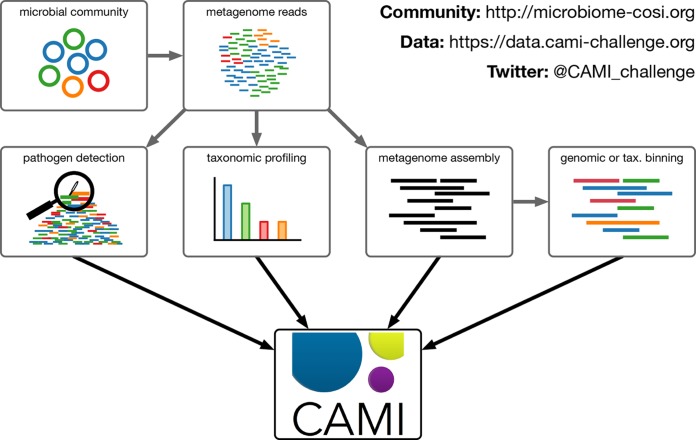
CAMI will provide data sets representing different environments and again offer metagenome assembly, profiling, and binning challenges, as well as a new pathogen detection challenge, which mimics an emergency diagnostic situation in the clinic.

Currently, CAMI is preparing for a second round of challenges, tentatively planned to open later this year. CAMI will provide data sets representing different environments and again offer assembly, taxonomic and genomic binning, as well as taxonomic profiling challenges (Fig. 1). Two multisample “toy” data sets representing microbial communities from different human body sites and from mouse gut are already provided to allow participants to prepare for the challenges (https://data.cami-challenge.org/participate). These practice data sets are generated from known genomes, and therefore reference-based methods (e.g., using genome databases for their analysis) might perform better here than for real shotgun metagenomic data, where a substantial portion of microbial community members have not been sequenced (3, 4). The second CAMI challenge data sets will therefore again include new genomes from taxa (at different evolutionary distances) not found in public databases. Furthermore, a new focus will be on establishing the value of long sequencing reads for microbiome research, with data sets providing both long- and short-read data. Lastly, a clinical pathogen discovery challenge will be offered, mimicking an emergency diagnostic situation in the clinic.

We invite everyone to join the CAMI effort and participate in the upcoming challenges!
